# Higher niacin intakes improve the lean meat rate of Ningxiang pigs by regulating lipid metabolism and gut microbiota

**DOI:** 10.3389/fnut.2022.959039

**Published:** 2022-10-06

**Authors:** Zhaobin Wang, Xianglin Zeng, Cheng Zhang, Qianqian Wang, Weidong Zhang, Junyan Xie, Jiashun Chen, Qin Hu, Qiye Wang, Huansheng Yang, Yulong Yin

**Affiliations:** ^1^Hunan Provincial Key Laboratory of Animal Intestinal Function and Regulation, Hunan International Joint Laboratory of Animal Intestinal Ecology and Health, Laboratory of Animal Nutrition and Human Health, College of Life Sciences, Hunan Normal University, Changsha, China; ^2^Key Laboratory of Subtropical Agro-ecological Processes, Hunan Provincial Engineering Research Center for Healthy Livestock and Poultry Production, Hunan Provincial Key Laboratory of Animal Nutritional Physiology and Metabolic Process, Scientific Observing and Experimental Station of Animal Nutrition and Feed Science in South-Central, Ministry of Agriculture, Institute of Subtropical Agriculture, Chinese Academy of Sciences, Changsha, China; ^3^National Center of Technology Innovation for Synthetic Biology, Tianjin Institute of Industrial Biotechnology, Chinese Academy of Sciences, Tianjin, China; ^4^Anyou Biotechnology Group Co., Ltd., Taicang, China

**Keywords:** niacin, Ningxiang pig, lipid metabolism, colon microbiota, LC-MS metabolomics

## Abstract

As one of the local pig breeds in China with a high fat rate, improving the lean meat rate of Ningxiang pigs through nutritional intervention is an urgent issue to be solved. As an important feed additive, niacin plays an important role in lipid metabolism. The purpose of this study was to investigate the regulation and mechanism of niacin on fat deposition in Ningxiang pigs. Thirty-four Ningxiang pigs (53.34 ± 2.78 kg) were randomly divided into two groups with five replicates each, with three to four Ningxiang pigs per replicate. The control group was fed a basal diet (contained 22 mg/kg niacin), and the experimental group was fed the same diet supplemented with an additional 100 mg/kg of niacin. The experimental period lasted 60 days. One Ningxiang pig was selected for slaughter sampling for each replicate. This study found that lean meat percentage of Ningxiang pigs in the experimental group was significantly increased (*P* < 0.05), accompanied by a significant decrease in fat percentage (*P* < 0.05). 16S rRNA sequencing analysis found an abundance of *Streptococcus* in the experimental group (*P* < 0.05), along with significantly decreased levels of *Lactobacillus* (*P* < 0.05). The changes in some OTUs belonging to *Firmicutes, Bacteroidota*, and *Actinobacteriota* were closely related to the changes in the fat rate and lean meat rate of Ningxiang pigs (*P* < 0.05). LC–MS metabolomics analysis found that about 43.75% of the differential metabolites were related to lipids and lipid-like molecules in the liver (*P* < 0.05). Spearman's correlation analysis showed correlations between the carcass traits, microbiota, and liver metabolites. In conclusion, niacin improves lean meat percentage and reduces fat deposition by regulating lipid metabolism and gut microbiota composition in Ningxiang pigs.

## Introduction

Ningxiang pig, a fatty-type pig breed indigenous to China, possesses the characteristics of a high reproductive rate, tolerance to rough feeding, gentle temperament, strong adaptability, and high meat quality during the feeding process (1). Compared with the current popular ternary hybrid pigs, Ningxiang pigs have obvious characteristics of high fat percentage and low lean meat percentage (2). With an increase in domestic income and consumption levels, pork consumption concepts will need to change accordingly. As such, the pork consumption market will develop keep in line with diversification, quality, and branding. Here, indigenous fatty-type breeds will usher in new development spaces and consumer markets. Therefore, it is of great significance to improve the carcass quality of Ningxiang pigs and reduce the fat rate through nutritional intervention.

Niacin (nicotinic acid), also called vitamin B3, is an essential water-soluble vitamin in pig diets (3, 4). It is the precursor of the cellular metabolic coenzymes nicotinamide adenine dinucleotide (NAD^+^) and nicotinamide adenine dinucleotide phosphate (NADP^+^). It participates in various redox reactions in animals in the form of NAD^+^ or NADP^+^ and plays an important role in lipid metabolism (5, 6). Adebowale et al. (7) found that high-dose niacin supplementation significantly reduced low-density lipoprotein and total cholesterol levels in turkey serum and meat. Another study also found that high doses of niacin inhibited lipid accumulation in rabbit hepatocytes (8). In addition to direct dietary supplements, niacin can also be synthesized from excess tryptophan from animals (9). In recent years, many reports have also clarified the physiological effects of niacin, such as maintaining antioxidant states, regulating energy production and metabolism, alleviating inflammation, and regulating blood lipid production (10–12). Niacin deficiency causes allergic reactions in animals, mainly manifested as enteritis, diarrhea, dermatitis, etc. (13). At present, niacin has been widely used to treat dyslipidaemia and atherosclerotic diseases (14). In addition, the latest research also pointed out that niacin has a significant effect on improving the intestinal health of weaned piglets (15).

Niacin is an important adipose regulator and has a good effect on animal growth and development, but its application in Ningxiang pigs is rarely reported. The purpose of this study was to focus on the effects of niacin on growth performance, fat metabolism, gut microbes, and meat quality and provide guidance for the healthy breeding of Ningxiang pigs.

## Materials and methods

### Animals and experimental design

This experiment was carried out at Hunan ChuWeiXiang Agriculture and Animal Husbandry Co., Ltd., Ningxiang, China. Thirty-four castrated Chinese Ningxiang pigs weighing 53.34 ± 2.78 kg were randomly divided into two groups, each with five repetitions, each repetition with three to four Ningxiang pigs. The control group (Control) was fed a corn–soybean meal basal diet (provided by Anyou Biotechnology Group Co., Ltd., Taicang, Jiangsu, China, which contained 22 mg/kg niacin), and the experimental group (VB3) was fed the same basal diet supplemented with an additional 100 mg/kg of niacin. The combination was mixed well with a mixer to ensure that the niacin was evenly distributed in the basal diet. The nutritional level of the diet (Table 1) met the Pig Nutrient Requirements of China (GB/T 39235-2020). Niacin products were purchased from Sangon Biotech (Shanghai) Co., Ltd., Shanghai, China, with a purity of 99%. Feeds and water were provided *ad libitum* throughout the experimental period. The daily management and vaccination of the pig farm were carried out in accordance with the company's normal procedures. The feed consumption of Ningxiang pigs in each pen was recorded daily. The feed intakes were aggregated throughout the experiment, and the pigs were weighed monthly. The average daily feed intake (ADFI), average daily gain (ADG), and ratio of daily feed intake to daily gain (F/G) were calculated according to the methods recommended by Wang et al. (16). After the experiment, one medium-weight Ningxiang pig was selected from each pen and euthanized according to Yan et al. (17). Using a sterile scalpel and surgical scissors, the abdominal cavity of the pig was opened, and the intestine, chyme, and liver samples were extracted.

**Table 1:** Basic diet composition and nutritional level of Ningxiang pigs, as-fed basis.

**Ingredient**	**Content**
Corn, %	42.31
Barley, %	15.00
Paddy, %	10.00
Rice bran, %	12.50
43% Soybean meal, %	12.40
Bread scraps, %	4.00
Stone powder, %	1.07
Calcium hydrogen phosphate, %	0.52
Sodium chloride, %	0.50
70% L-Lysine sulfate, %	0.20
Premix^a^, %	1.5
Total	100
**Calculated nutritional level**	
CP, %	12.83
NE, kcal/kg	2,266.40
Lys^b^, %	0.60
Met + Cys^b^, %	0.62
Trp^b^, %	0.20
Thr^b^, %	0.71
Ca, %	0.60
Total *P*, %	0.56
Available *P*, %	0.23

a Supplied per kilogram of diet: Fe, 90 mg; Cu, 3.5 mg; Mn, 3 mg; Zn, 55 mg; I, 0.15 mg; Se, 0.25 mg; vitamin A, 1350 IU; vitamin D_3_, 160 IU; vitamin E, 15 IU; vitamin K, 0.5 mg; vitamin B_12_, 6 μg, riboflavin, 3.5 mg; D-pantothenic acid, 10.5 mg; biotin, 0.1 mg; folic acid, 0.5 mg; choline, 0.45 g.

b Standard ileal digestible.

### Carcass traits and meat quality determination

According to the requirements of Technical Specification for the Determination of Carcass Traits of Pigs (NY/T 825-2004), the carcass traits of Ningxiang pigs were determined. The left carcass was stripped of skin, bone, fat, and lean meat. When stripping, the intermuscular fat was counted as lean meat, the skin was counted as fat, the cartilage and tendon were counted as lean meat, and the lean meat on the bones was stripped clean. The loss during peeling was no >2%. Ningxiang pigs were slaughtered, and their carcasses were weighed after removing the hair, head, tail, hooves, and internal organs. The carcass oblique length (the oblique length from the front edge of the pubic symphysis to the junction of the first rib and the sternum) and the carcass straight length (the straight length from the anterior border of the pubic symphysis to the anterior border of the first cervical vertebra) were measured. The average fat thickness at the thickest part of the shoulder, last rib, and lumbar–sacral junction of the right dorsal midline of the right carcass was measured with a vernier caliper. The longissimus dorsi muscle was cut vertically at the last rib of the left carcass, the width and height of the cross section were measured, and the longissimus dorsi muscle area was calculated according to the formula: width^*^height^*^0.7. The hip weight of the left leg, cut vertically between the penultimate lumbar vertebrae and the penultimate lumbar vertebra, was weighed, and the ratio was calculated. The skin, leaf lard, lean meat, and fat were weighed separately, and their ratios were calculated.

According to NY/T821-2004, the collection of muscle samples was completed within 1–2 h of cessation of respiration in pigs for meat quality determination. The longissimus dorsi muscle at the junction of the waist and sacrum was taken, and the flesh color was measured according to the requirements of the colorimeter. The penultimate 1–2 longissimus dorsi of the thoracic vertebra was taken, cut into small pieces after removing the peripheral sarcolemma of the meat samples, then placed in a meat grinder, and ground into minced meat. The pH values of Ningxiang pigs at 45 min (pH_45min_) and 24 h (pH_24h_) after slaughter were measured with a hand-held pH meter. Within 1–2 h of cessation of respiration, the longissimus dorsi of the penultimate 3–4 thoracic vertebral segment was taken, the peripheral sarcolemma of the muscle was removed, and two strips of about 4 cm × 4 cm × 4 cm were trimmed along the direction of the muscle fibers. Each strip of meat was weighed before hanging. One end of the meat strip was pierced with a hanging hook and placed in the center of the food bag to avoid contact between the meat sample and the bag. The bag was then placed in a refrigerator at 2–4°C for 48 h; then, the muscle was taken out from the refrigerator. The surface moisture was absorbed with absorbent paper, and the sample was weighed. The dripping loss was calculated according to the formula: (weight before hanging – weight after hanging)/weight before hanging × 100.

### Bacterial composition of the colonic microbiota

Microbial diversity of colonic contents of Ningxiang pigs was analyzed by 16S rRNA sequencing technology. DNA was extracted from the colonic contents of Ningxiang pigs using a fecal genomic DNA extraction kit (TIANGEN, Beijing, China) according to the manufacturer's instructions. DNA quality and concentration were assessed by using 1% agarose gel electrophoresis and a NanoDrop 2000 spectrophotometer, respectively. The colonic contents of Ningxiang pigs were randomly selected for pre-experiment to ensure that most of the samples could amplify products with appropriate concentrations in the lowest cycle number and to fully prepare for the formal experiment of all samples. After the pre-experiment was completed, the formal PCR (ABI GeneAmp^®^ 9700, Applied Biosystems, USA) test used TransStart FastPfu DNA polymerase (AP221-02, TransGen Biotech, Beijing, China) and universal primer 338F_806R (forward: 5′-ACTCCTACGGGAGGCAGCAG-3′ and reverse: 5′-GGACTACHVGGGTWTCTAAT-3′), and the reaction system was 20 μL (5 × FastPfu buffer, 4 μL; 2.5 mM dNTPs, 2 μL; 5 μM forward primer, 0.8 μL; 5 μM reverse primer, 0.8 μL; FastPfu polymerase, 0.4 μL; BSA, 0.2 μL; template DNA, 10 ng; make up with ddH_2_O to 20 μL). The amplification programme consisted of one cycle of 95°C for 3 min, followed by 40 cycles of 95°C for 30 s, 55°C for 30 s, and 72°C for 45 s, and finally one cycle of 72°C for 10 min.

The PCR products were purified by using a gel extraction kit. DNA libraries were constructed using the TruSeqTM DNA Sample Prep kit (Illumina, California, USA). The constructed library was sequenced by Shanghai Majorbio Bio-Pharm Technology Co., Ltd., Shanghai, China. The PE reads obtained by MiSeq sequencing were used for paired-end sequence splicing using FLASH software (version 1.2.11, https://ccb.jhu.edu/software/FLASH/index.shtml) and QIIME software (version 1.9.1, http://qiime.org/install/index.html) to perform quality filtering of raw reads. The clean reads obtained after execution were OTU clustered using UPARSE software (version 7.0.1090, http://www.drive5.com/uparse/). Alpha diversity analysis was performed using mothur (version 1.30.2, https://www.mothur.org/wiki/Download_mothur). The results of the alpha diversity analysis were presented in Simpson's index, ACE index, Chao1 index, the observed number of OTUs (Sobs), and sequencing depth.

### Liver sample preparation, untargeted metabolomics analysis, quality control, and data processing

After weighing 50 mg of each Ningxiang pig liver sample, 400 μL of the extraction solution containing the internal standard (methanol–acetonitrile volume ratio of 1:1 and the internal standard concentration of 2 μg/mL) was added, and a high-throughput tissue grinder was used at low temperature (60 Hz, −20°C) for pulverization. The extracted samples were then placed at −20°C for 30 min, subjected to vortex mixing and sonication at 40 kHz, and left at 4°C for 30 min. Then, the samples were centrifuged at 12,000×*g* for 15 min at 4°C, and the supernatant was taken for testing in the LC–MS system.

LC–MS was performed at Shanghai Majorbio Bio-Pharm Technology Co., Ltd., Shanghai, China. The LC–MS analysis platform in this study was the AB SCIEX UPLC-TripleTOF system. The chromatographic column was a UPLC BEH Amide column (1.7 μm × 21 mm × 100 mm, Waters, Milford, USA). Mobile phase A was HPLC grade water (containing 0.1% formic acid), and mobile phase B was acetonitrile (containing 0.1% formic acid). The proportions of the mobile phases varied over time: 95% A and 5% B for 3 min, 80% A and 20% B for 6 min, 5% A and 95% B for 4 min, and 95% A and 5% B for 3 min. The column temperature was 40°C, the flow rate was 0.4 mL/min, and the injection volume was 20 μL. Mass spectrometry data of liver metabolites were acquired using positive and negative ion scanning modes. The collision energy was 30 eV, the electrospray voltage was 4,500 V, the atomization air pressure was 60 Psi, the auxiliary air pressure was 60 Psi, the air curtain air pressure was 35 Psi, and the ion source temperature was 500°C. The mass spectrometry scan range was 70–1,050 m/z.

To ensure the stability of the analytical system, all test samples were pooled as quality control samples. During instrumental analysis, a quality control sample was inserted every 8–10 samples. The relative standard deviation of the internal standard was <30%, indicating that the stability and repeatability of the analytical system were good (18).

Raw data were subjected to peak detection and alignment using Progenesis QI 2.3 software (Waters, Milford, USA). In order to better analyse the data, a series of pre-processing steps need to be performed on the original data, including filtering of missing values in the original data, missing value recoding, data normalization, QC verification (RSD < 30%), and data transformation (log10 transformation). According to the parameters such as retention time and mass-to-charge ratio (m/z), the metabolites with differences were identified by comparison in the Human Metabolome Database (HMDB, http://www.hmdb.ca/). Principal component analysis (PCA) was performed using ropls (R packages, Majorbio, Shanghai, China).

### Statistical analysis

First, it was checked whether the normal distribution and homogeneity of variance of growth performance, carcass traits, and meat quality between the two groups met the requirements of subsequent analysis. The significance of the difference between the two groups was then compared by Student's *t*-test. *P* < 0.05 indicated a significant difference. 0.05 < *P* < 0.10 indicated a trend difference. *P* > 0.10 indicated no significant difference. Based on the community abundance data in the samples, the Student's *t*-test was used to detect species that exhibited differences in abundance in the two groups of microbial communities, and a hypothesis test was performed to evaluate the significance of the observed differences. The microbial taxa that had a significant effect on the differences between groups were tested by LEfSe linear discriminant analysis (LDA > 4). A heatmap of correlations between OTUs and carcass traits (fat and lean meat rate) of Ningxiang pigs was analyzed by Spearman's correlation (R, version 3.3.1, pheatmap package). To initially screen the differential metabolites, an analysis of variance (ANOVA, *P* < 0.05) was performed using the R packages. These differential metabolites were screened by variable importance in projection (VIP > 1) in partial least squares discriminant analysis (PLS-DA). PLS-DA data conversion was performed by Pareto conversion. The confidence level was 0.95, and 200 permutation tests were performed. The abundance of metabolites was detected by Student's *t*-test. The pathways involved in the differential metabolites were identified in the Kyoto Encyclopedia of Genes and Genomes (KEGG, http://www.genome.jp/kegg/). Spearman's correction analysis was performed to examine the correlations between microbial OTUs (which were strongly associated with the fat rate and lean meat rate, numbering 13 OTUs in total) and hepatic metabolites.

## Results

### Growth performance, carcass traits, and meat quality

The supplementation of 100 mg/kg niacin in the diet had no significant effect on the body weight, ADG, ADFI, and F/G of Ningxiang pigs (Table 2, *P* > 0.05). In terms of carcass characteristics, the leg-to-hip ratio tended to decrease in the experimental group (Table 3, *P* = 0.058), while bone rates tended to increase (*P* = 0.058) and fat percentages decreased significantly (*P* = 0.002). At the same time, the percentage of lean meat increased significantly (*P* = 0.021). However, there were no significant differences in slaughter rate, skin rate, eye muscle area, skin thickness, backfat thickness, carcass straight length, and carcass oblique length between the two groups (*P* > 0.05). In terms of meat quality, the addition of niacin did not significantly affect the drip loss, meat color, and pH_45min_ or pH_24h_ of the longissimus dorsi of Ningxiang pigs (Table 4, *P* > 0.05).

**Table 2:** Effects of diet supplementation with niacin on growth performance of Ningxiang pigs^a^.

**Items**	**Control**	**VB3**	**SEM**	**T-test**
Initial body weight, kg	53.57	53.11	2.78	0.940
Final body weight, kg	79.68	76.37	3.48	0.661
ADG, g/d	450.29	401.01	19.05	0.213
ADFI, g/d	2199.89	2105.14	77.14	0.571
F:G	4.94	5.25	0.16	0.375

a Control, Ningxiang pigs were fed a basal diet. VB3, an additional 100 mg/kg of niacin was added to the basal diet of Ningxiang pigs. ADG, average daily gain; ADFI, average daily feed intake; F/G, ratio of ADFI to ADG; SEM, standard error of the mean.

**Table 3:** Effects of dietary supplementation of niacin on carcass traits of Ningxiang pigs^a^.

**Items**	**Control**	**VB3**	**SEM**	**T-test**
Slaughter rate, %	75.98	76.16	0.28	0.762
Leg-to-hip ratio, %	26.37	24.05	0.63	0.058
Leaf lard rate, %	4.71	5.31	0.30	0.354
Skin rate, %	12.65	13.79	0.48	0.260
Bone rate, %	15.03	16.59	0.42	0.058
Fat rate, %	38.26	33.30^**^	0.97	0.002
Lean meat rate, %	34.07	36.33^*^	0.53	0.021
Dorsal muscle area, mm^2^	1405.90	1528.13	74.09	0.442
Skin thickness, mm	5.81	6.17	0.32	0.610
Backfat thickness, mm	42.75	37.51	2.36	0.292
Carcass straight length, cm	81.60	84.30	1.45	0.381
Carcass oblique length, cm	68.50	70.40	1.07	0.408

a Control, Ningxiang pigs were fed a basal diet. VB3, an additional 100 mg/kg of niacin was added to the basal diet of Ningxiang pigs. SEM, standard error of the mean.

* P < 0.05;

** P < 0.01.

**Table 4:** Effects of dietary supplementation of niacin on meat quality of Ningxiang pigs^a^.

**Items**	**Control**	**VB3**	**SEM**	**T-test**
Drip losses, %	13.96	12.16	1.58	0.599
pH_45min_	5.65	5.72	0.08	0.675
pH_24h_	5.64	5.62	0.06	0.880
L^*^	46.91	43.82	1.22	0.224
a^*^	11.69	10.75	0.52	0.389
b^*^	7.18	6.19	0.40	0.237

a Control, Ningxiang pigs were fed a basal diet. VB3, an additional 100 mg/kg of niacin was added to the basal diet of Ningxiang pigs. SEM, standard error of the mean.

### Colonic microbiota

**Figure 1:**
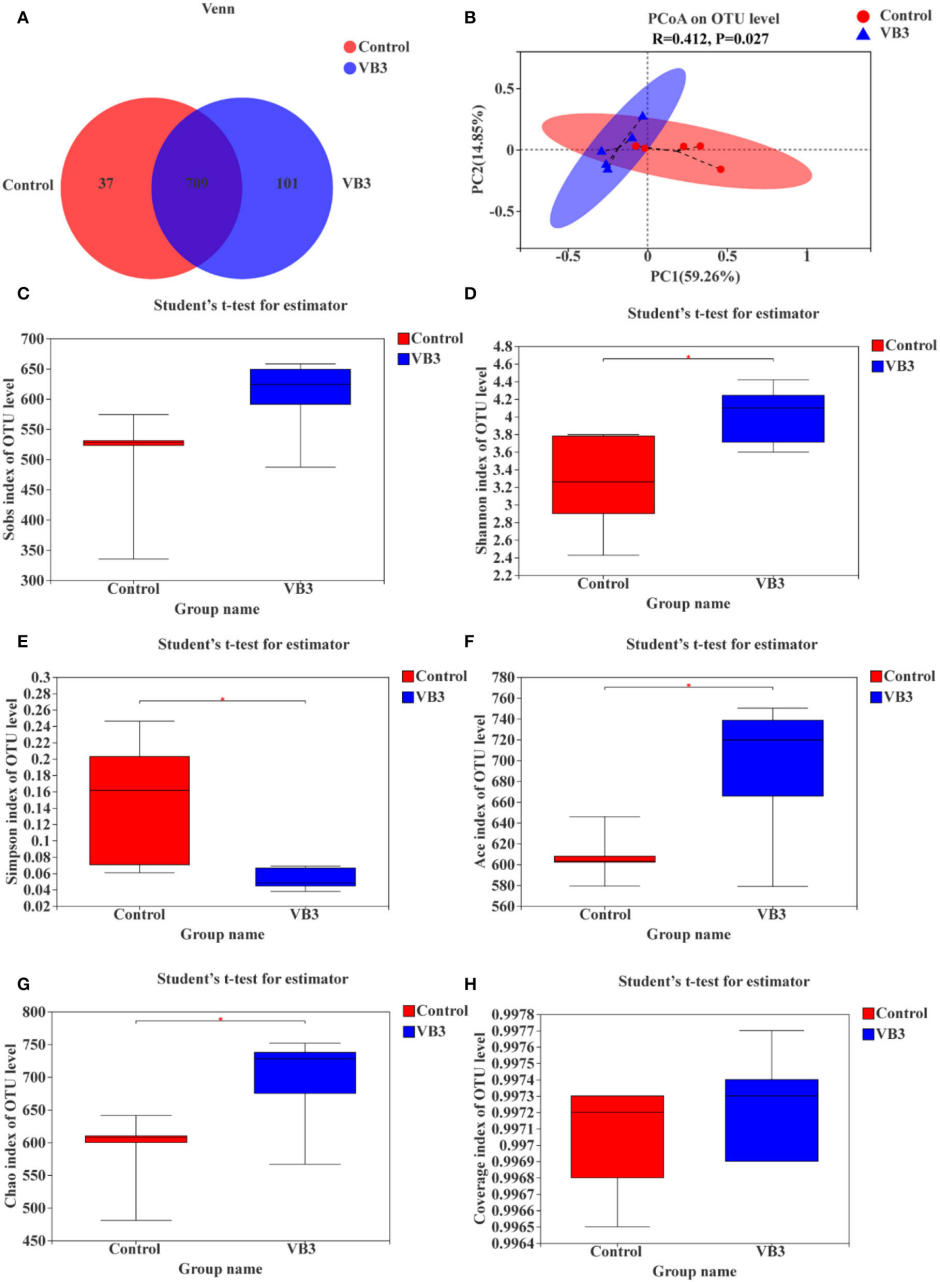
OTUs analysis, β-diversity estimation, and α-diversity analysis between the experimental group (VB3) and control group (Control). **(A)** Venn diagram based on OTU. **(B)** PCoA for β-diversity estimation. **(C–H)** Box plot of alpha diversity [**(C)** observed species number; **(D)** Shannon's index; **(E)** Simpson's index; **(F)** ACE index; **(G)** Chao1 index; **(H)** coverage index]. *P < 0.05.

A total of 534,662 optimized sequence reads were obtained, with 221,735,375 bases and an average sequence length of 415 bp. The study found that the colon produced a total of 847 OTUs, of which 709 overlapped. The number of OTUs in the experimental group (810 OTUs) was higher than that in the control group (746 OTUs) (Figure 1A). PCoA showed that there was a significant difference between the two groups (*P* = 0.027, Figure 1B). The colonic microbiota in the niacin and control groups were divided into two distinct clusters. The contribution rates of PCoA1 and PCoA2 to the total variance were 59.26 and 14.85%, respectively. Furthermore, it was found by the alpha diversity index test that when compared with the control group, the Shannon (Figure 1D), ACE (Figure 1F), and Chao1 (Figure 1G) indices in the experimental group were significantly increased (*P* < 0.05), while the Simpson index (Figure 1E) was significantly decreased (*P* < 0.05). These all reflect the significant increase in the total number of species and community diversity of colonic microorganisms in the experimental group. The observed number of OTUs (Sobs, Figure 1C) and sequencing depth (Figure 1H) were not significantly different (*P* > 0.05).

**Figure 2:**
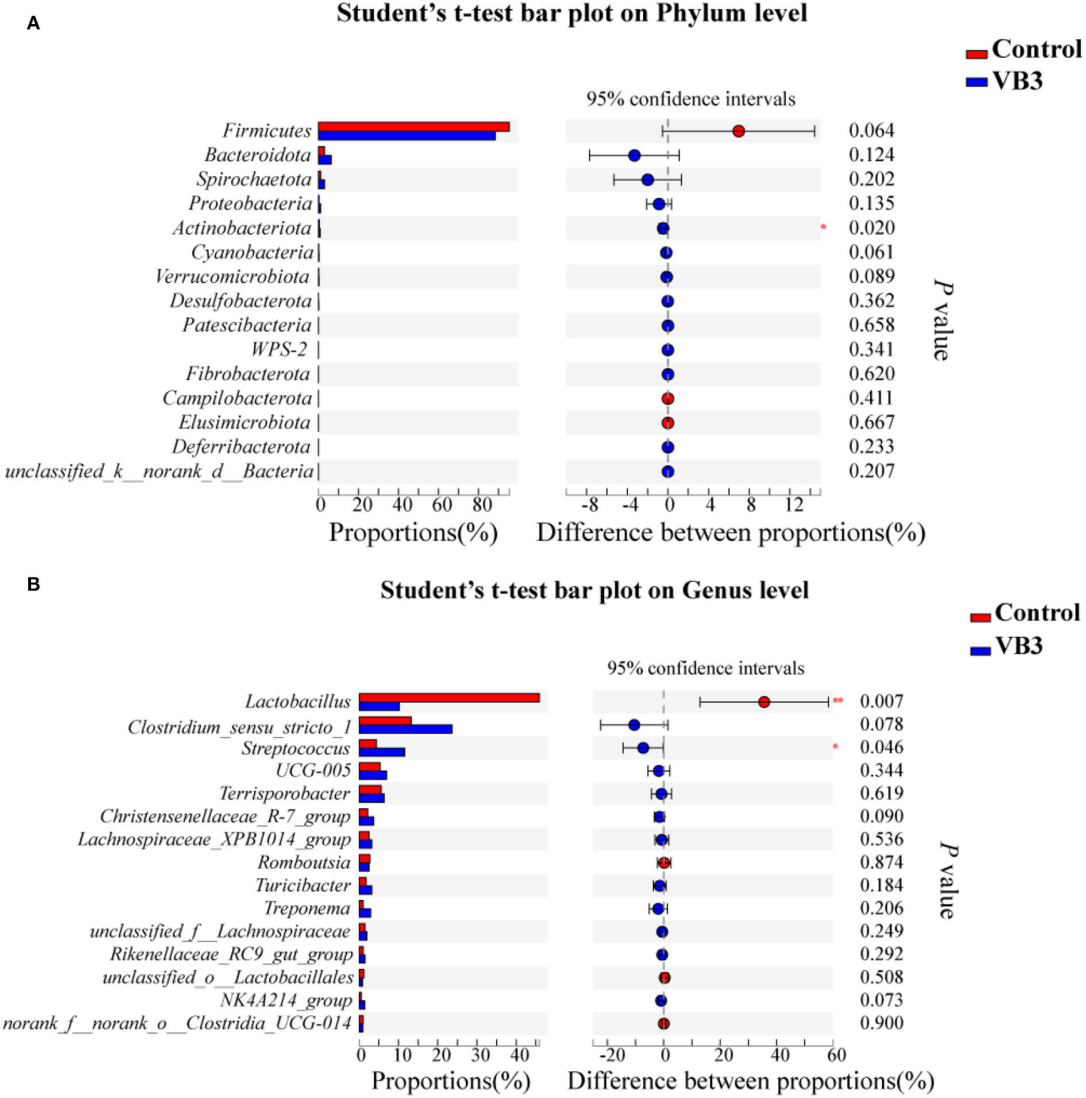
Relative abundance of bacteria community in the experimental group (VB3) and control group (Control) at phylum **(A)** and genus **(B)** levels. *P < 0.05; **P < 0.01.

**Figure 3:**
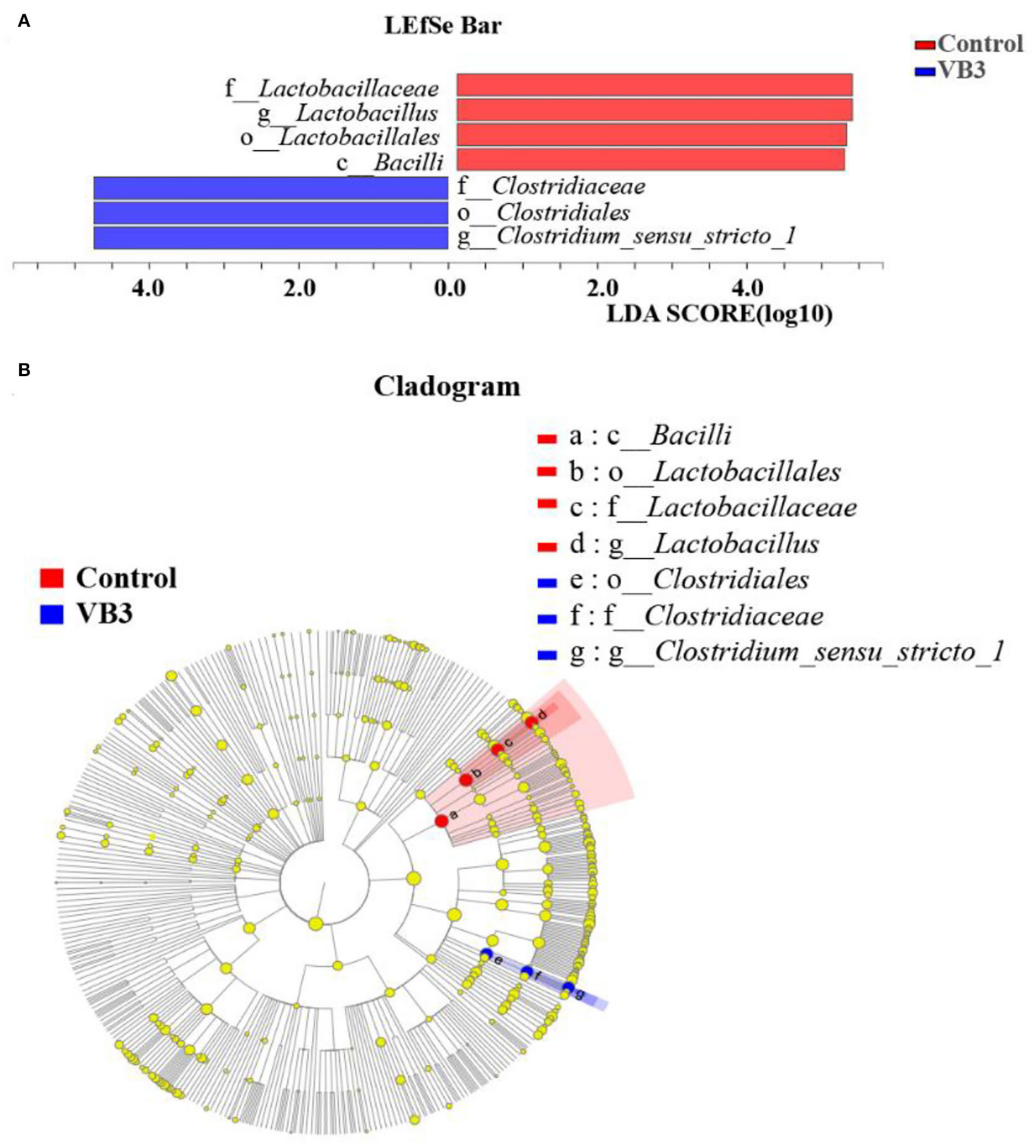
LEfSe analysis between the experimental group (VB3) and control group (Control). Red, control group. Blue, experimental group. **(A)** Histogram of the results of LEfSe between the experimental and control groups and their respective effect sizes; *P*-value < 0.05 considered significant. **(B)** Cladogram showing taxonomic representation of differences between the experimental and control groups.

Figure 2 shows the microbial composition at the top 15 most abundant phylum and genus levels in the colonic contents. At the phylum level, the abundance of *Actinobacteriota* was significantly increased in the experimental group (*P* < 0.05). At the genus level, the abundance of *Lactobacillus* decreased significantly in the experimental group, while the abundance of *Streptococcus* increased significantly (*P* < 0.05). LEfSe multilevel species difference discriminant analysis found that the abundance of four taxa (*Bacilli* class, *Lactobacillales* order, *Lactobacillus* family, and *Lactobacillaceae* genus) in the control group was significantly increased (*P* < 0.05). The abundance of five taxa (*Clostridiales* order, *Clostridiaceae* family, *Faecalibacterium* genus, *Clostridium_sensu_stricto_1* genus, and UBA1819 genus) in the experimental group was significantly increased (*P* < 0.05, Figure 3A). As shown in Figure 3B, compared with *Bacillus* (class) in the control group, the significantly different bacteria in the experimental group were mainly concentrated in *Clostridia* (class). These key phylotypes contributed to the differences in microbiota composition between the two groups.

### Metabolic profiles

**Figure 4:**
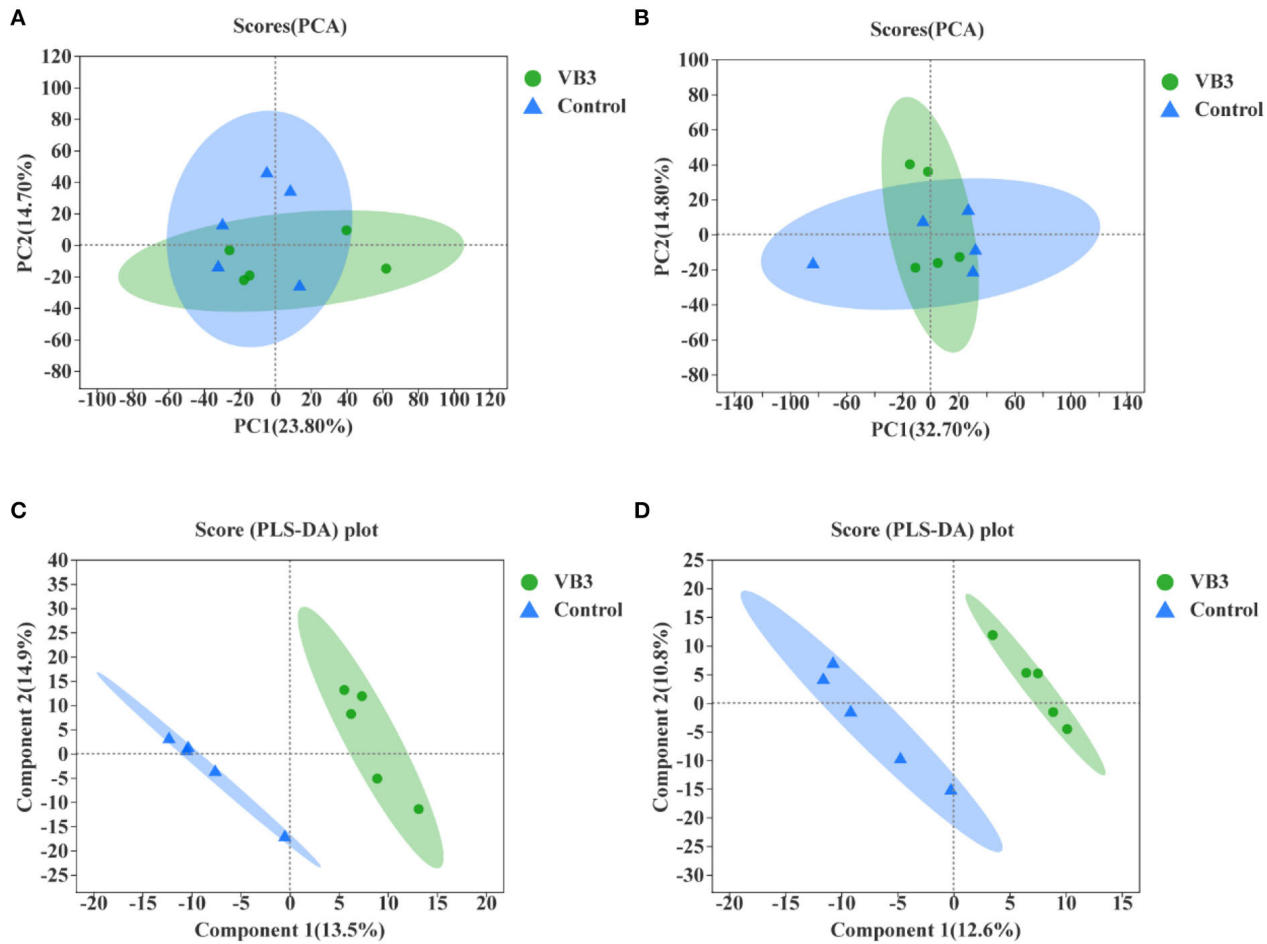
Principal component analysis (PCA) between the experimental group (VB3) and control group (Control) and partial least squares discriminant analysis (PLS-DA) between the experimental group (VB3) and control group (Control). **(A)** PCA based on positive ion table. **(B)** PCA based on negative ion table. **(C)** PLS-DA based on positive ion table. **(D)** PLS-DA based on negative ion table.

Through LC–MS and statistical software, 4,117 positive ion mass spectral peaks and 3,578 negative ion mass spectral peaks were extracted. In the positive ion mode, 394 metabolites have been identified, of which 293 were annotated to public databases such as HMDB and LIPID MAPS and 141 were annotated to the KEGG database. In the negative ion mode, 297 metabolites have been identified, of which 190 were annotated to public databases such as HMDB and LIPID MAPS and 112 were annotated to the KEGG database. The PCA of liver metabolic profile is shown in Figure 4. There was no significant difference in the hepatic metabolic profile between the experimental group and the control group in both positive and negative ion modes. By the PLS-DA, the data of the two groups were clearly distinguishable. There was no overlap in the data between the groups.

**Figure 5:**
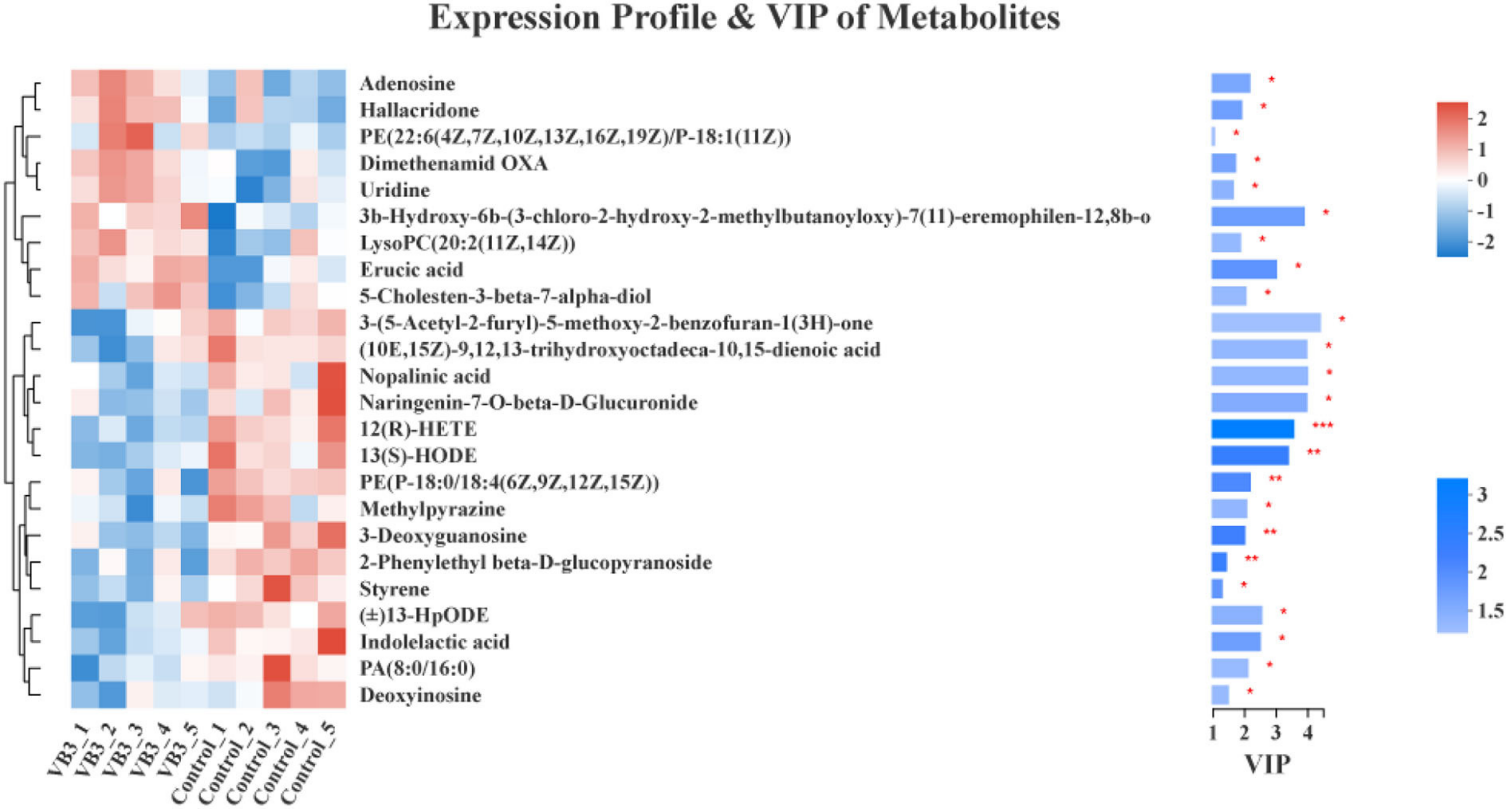
Differential metabolite cluster dendrogram between the experimental group (VB3) and control group (Control) and variable importance in projection (VIP) of metabolites between the experimental group (VB3) and control group (Control). *P < 0.05; **P < 0.01; ***P < 0.001.

**Figure 6:**
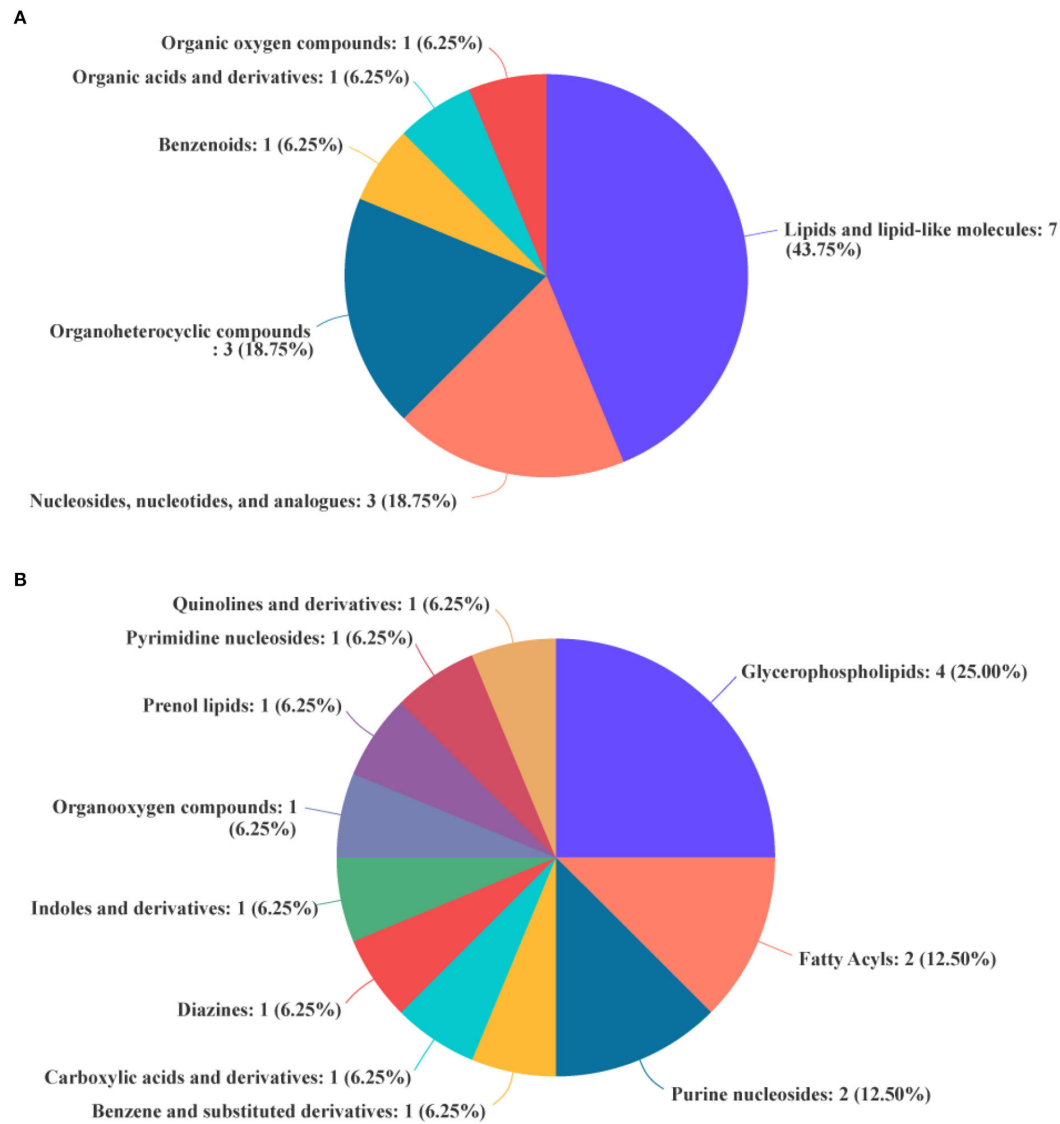
Biochemical categories of the differential metabolites identified between the experimental group (VB3) and control group (Control). **(A)** Superclass. **(B)** Class.

**Figure 7:**
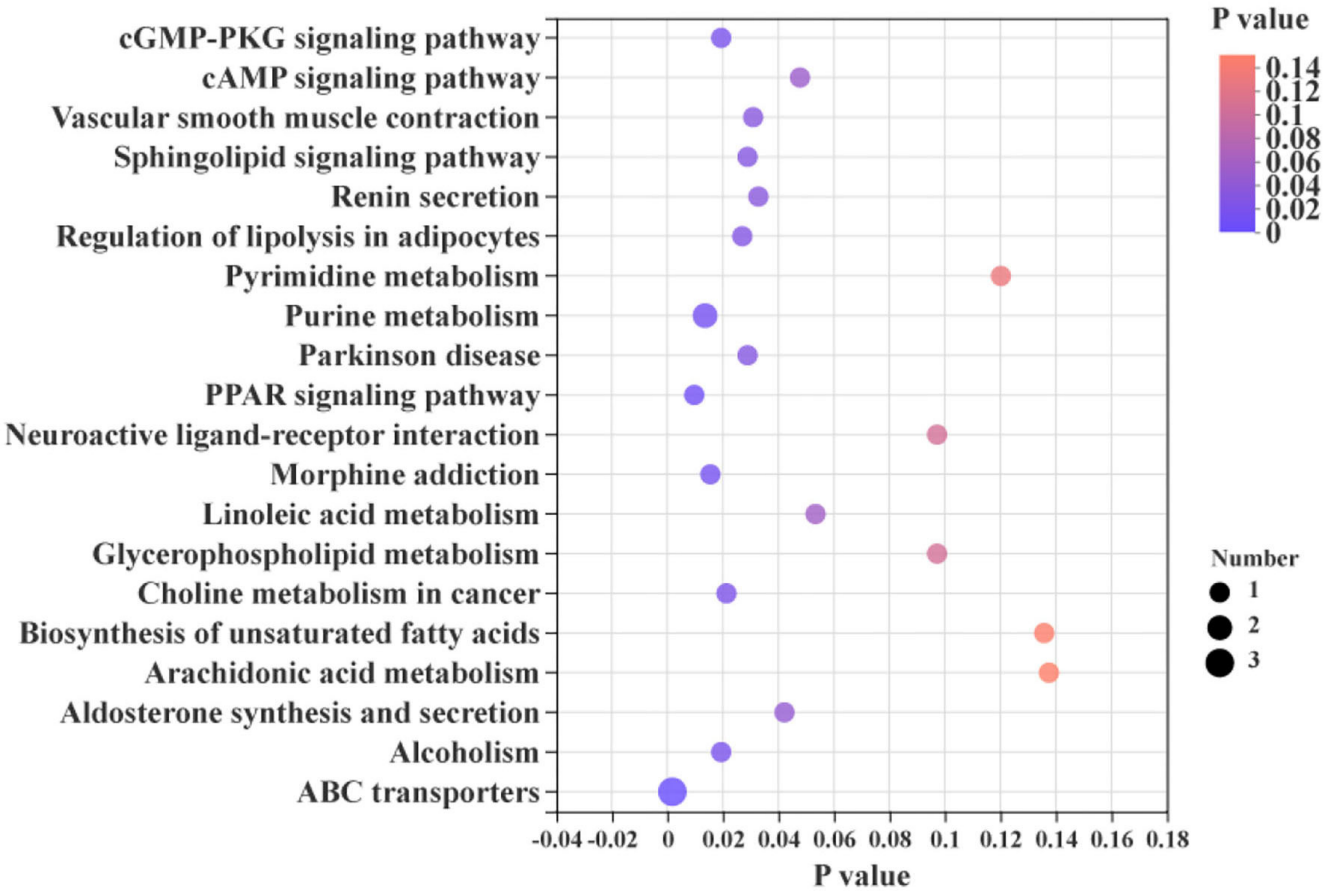
KEGG enrichment analysis between the experimental group (VB3) and control group (Control).

The differential metabolites in the livers of the two groups were screened, and a total of 24 differential metabolites were obtained (Figure 5). The HMDB compound classification showed that these metabolites were mainly divided into six superclasses and 11 classes (Figure 6). In the superclasses, the number of differential metabolites of lipids and lipid-like molecules was 7 [about 43.75%, including classes of glycerophospholipids (4), fatty acyls (2), and prenol lipids (1)]. Metabolic pathway analysis was performed for the differential metabolites in each liver subsequently. The study found that the treatment of niacin mainly had a significant effect on cGMP-PKG and cAMP signaling pathway, sphingolipid signaling pathway, regulation of lipolysis in adipocytes, purine metabolism, renin secretion, aldosterone synthesis and secretion, PPAR signaling pathway, and ABC transporters of Ningxiang pigs (Figure 7).

### Correlations between carcass traits, gut microbiota, and hepatic metabolites

**Figure 8:**
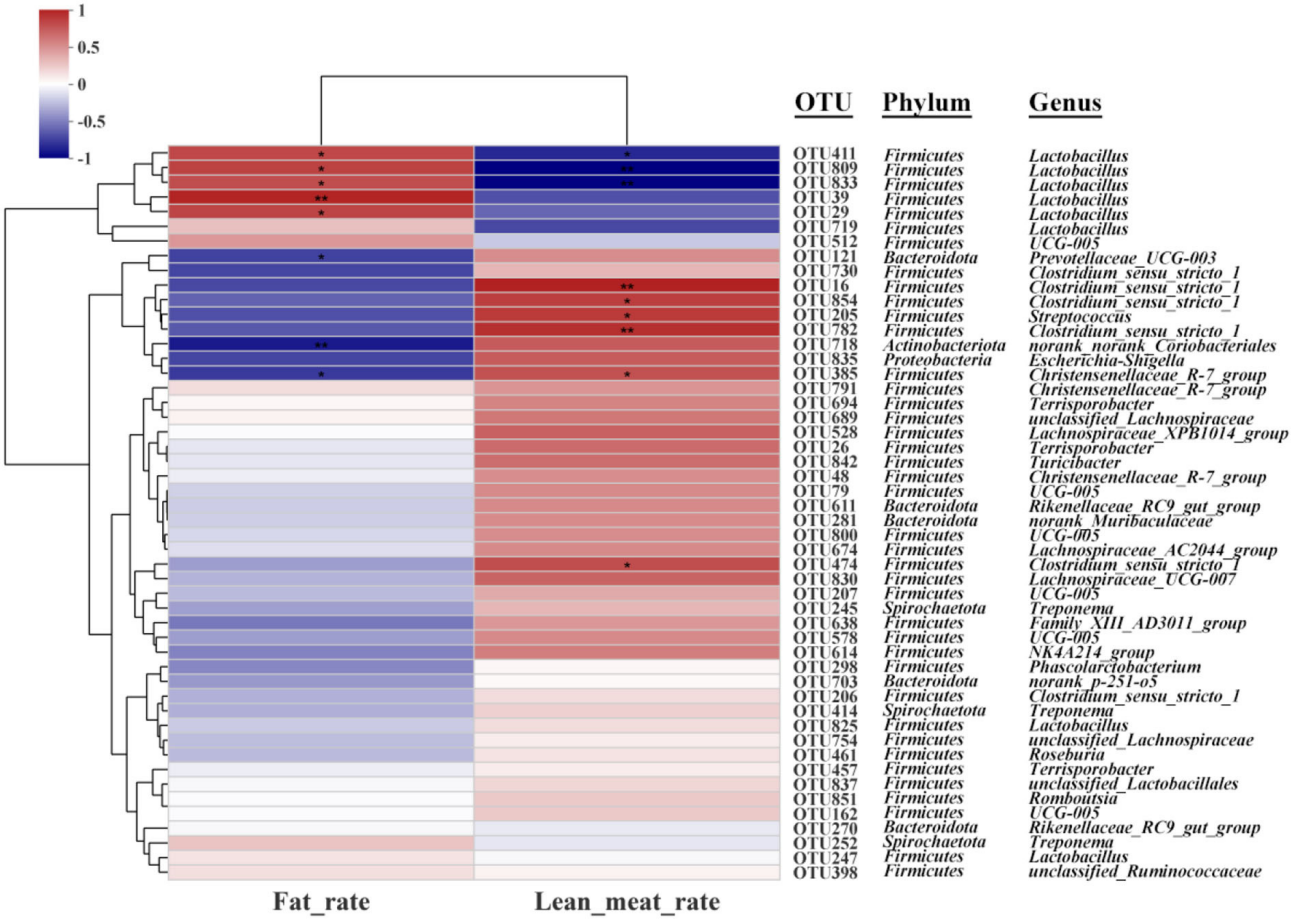
Spearman's correlation analysis between gut microbiota (OTU level) and carcass traits (fat rate and lean meat rate). *P < 0.05; **P < 0.01.

Spearman's correction analysis was performed to examine the correlations between carcass traits (fat and lean meat rate), the abundance of top 50 gut microbiota, and liver metabolites in Ningxiang pigs. The gut microbiota and carcass traits (fat and lean meat percentages) of Ningxiang pigs were analyzed by Spearman's correction analysis to explore the key microbiota that niacin encourages to reduce fat deposition in Ningxiang pigs (Figure 8). The results revealed that *Lactobacillus* (OTU411, OTU809, and OTU833) was positively correlated with the fat rate and negatively correlated with the lean meat rate. *Christensenellaceae_R-7_group* (OTU385) was negatively correlated with the fat rate and positively correlated with the lean meat rate. *Lactobacillus* (OTU29 and OTU39) was positively correlated with the fat rate. *Prevotellaceae_UCG_003* (OTU121) and *norank_norank_Coriobacteriales* (OTU718) were negatively correlated with the fat rate. *Clostridium_sensu_stricto_1* (OTU16, OTU474, OTU782, and OTU854) and *Streptococcus* (OTU205) were positively correlated with the lean meat rate.

**Figure 9:**
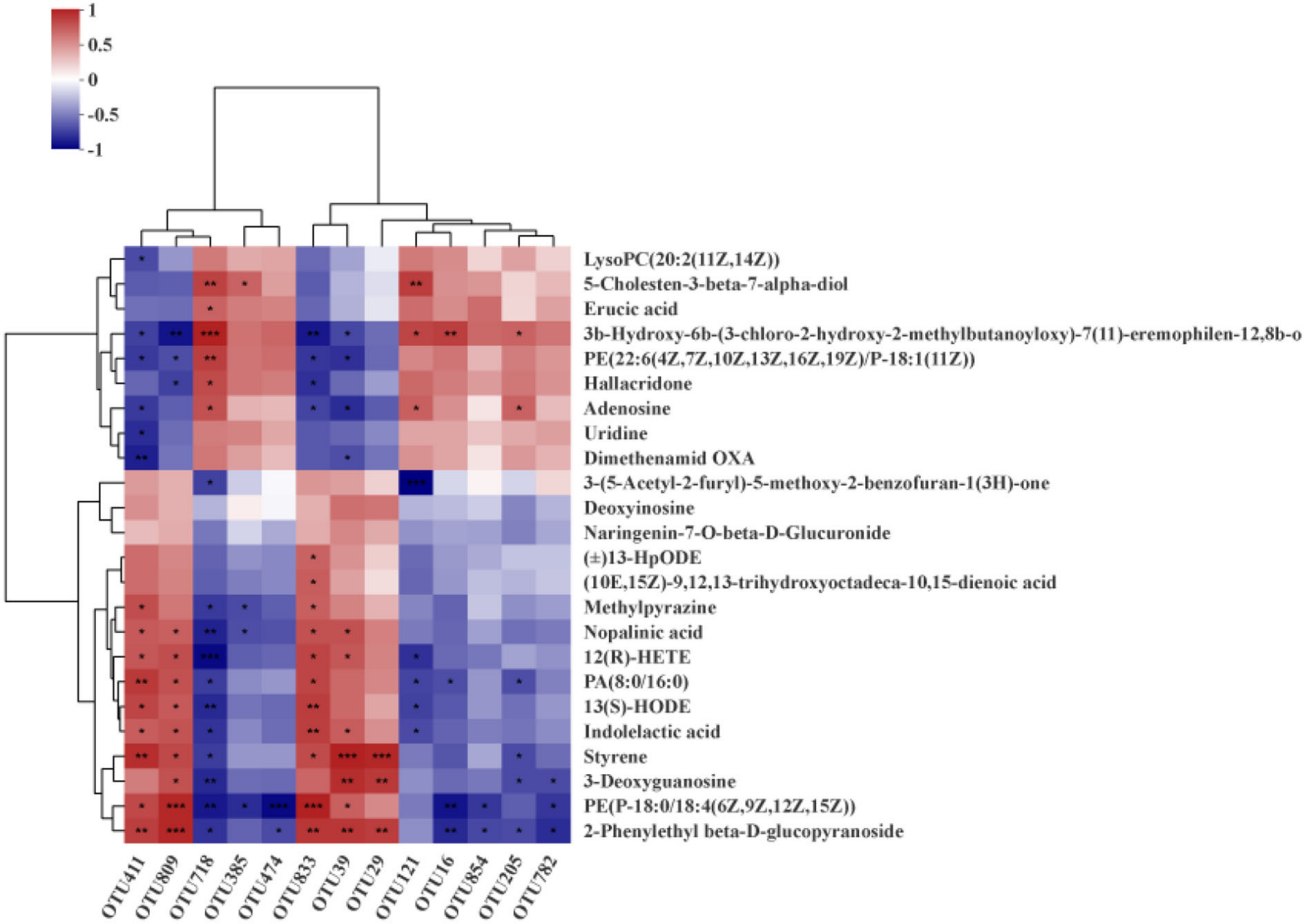
Spearman's correlation analysis between hepatic metabolites and gut microbiota (OTU level). *P < 0.05; **P < 0.01; ***P < 0.001.

Next, Spearman's correction analysis was performed to examine the correlations between the 13 microbial OTUs that were strongly associated with the fat rate and lean meat rate and hepatic metabolites (Figure 9). The results showed that about 12 metabolites (glycoside, glycerophosphoethanolamine, 3-deoxyguanosine, styrene, indolelactic acid, 13-hydroxyoctadecadienoic acid, hexadecanoate, tetraenoic acid, nopalinic acid, methylpyrazine, corchorifatty acid F, and hydroperoxyoctadecadienoic acid) were significantly positively correlated with *Lactobacillus* (OTU29, OTU39, OTU411, OTU809, and OTU833). Seven metabolites (lysophosphatidylcholine, methylbutanoate, phosphoethanolamine, hallacridone, adenosine, uridine, and dimethenamid OXA) were significantly negatively correlated with *Lactobacillus* (OTU29, OTU39, OTU411, OTU809, and OTU833). Methylbutanoate was significantly positively correlated with *Clostridium_sensu_stricto_1* (OTU16). Hexadecanoate, 3-deoxyguanosine, glycerophosphoethanolamine, and glycoside were significantly negatively correlated with *Clostridium_sensu_stricto_1* (OTU16, OTU474, OTU782, and OTU854). Methylbutanoate, 7-alpha-hydroxycholesterol, and adenosine were significantly positively correlated with *Prevotellaceae_UCG_003* (OTU121). Benzofuran, tetraenoic acid, hexadecanoate, 13-hydroxyoctadecadienoic acid, and indolelactic acid were significantly negatively correlated with *Prevotellaceae_UCG_003* (OTU121). Methylbutanoate and adenosine were significantly positively correlated with *Streptococcus* (OTU205). Hexadecanoate, styrene, 3-deoxyguanosine, and glycoside were significantly negatively correlated with *Streptococcus* (OTU205). 7-Alpha-hydroxycholesterol, erucic acid, methylbutanoate, phosphoethanolamine, hallacridone, and adenosine were significantly positively correlated with *norank_norank_Coriobacteriales* (OTU718). Methylpyrazine, nopalinic acid, tetraenoic acid, hexadecanoate, 13-hydroxyoctadecadienoic acid, indolelactic acid, styrene, 3-deoxyguanosine, glycerophosphoethanolamine, and glycoside were significantly negatively correlated with *norank_norank_Coriobacteriales* (OTU718). 7-Alpha-hydroxycholesterol was significantly positively correlated with *Christensenellaceae_R-7_group* (OTU385). Methylpyrazine, nopalinic acid, and glycerophosphoethanolamine were significantly negatively correlated with *Christensenellaceae_R-7_group* (OTU385).

## Discussion

As one of the important B vitamins, niacin is essential in pig diets. Several recent studies have shown that niacin plays an important role in regulating lipid metabolism (6, 19). As an excellent local fat pig breed in China, Ningxiang pig is characterized by a high fat rate and a low lean meat rate. This study is the first to report the application effect of niacin as a feed additive in Ningxiang pigs. Previous studies have shown that the addition of 110 mg/kg niacin in the diet has no significant effect on the growth performance of finishing pigs in two different growth environments (20). Similarly, two other studies showed no significant difference in growth performance in weaning and growing pigs with niacin supplementation (15, 21). However, there are also some studies that indicate that niacin increases G/F in piglets at weaning stage (22, 23). Different research findings provide new insights into niacin improving pig growth performance. This study found that additional supplementation of 100 mg/kg niacin did not change the growth performance of Ningxiang pigs at the 50–80-kg stage, which is somewhat inconsistent with previous research. The experimental subjects of this study were Ningxiang pigs in the fattening period. The niacin content (22 mg/kg) in the basal diet at this stage can fully meet the nutritional needs of the fattening period, and additional dietary niacin content will not have much impact on growth performance. This finding is consistent with the study by Real et al. (20). At the weaning stage, due to the influence of weaning stress, piglets urgently need to obtain nutrients from the outside world to relieve weaning stress. Here, additional supplementation of niacin will instead inform growth performance in a better direction. Thus, the effect of niacin on the growth performance of Ningxiang pigs needs to be further verified through larger-scale animal experiments.

Carcass traits are important economic indicators for evaluating the pig industry. In this study, an additional 100 mg/kg of niacin supplementation significantly increased lean meat and bone percentages and decreased fat percentage and leg-to-hip ratio in Ningxiang pigs. This also suggested that the additional supplemented niacin was mainly used for the growth of muscle and tissue in Ningxiang pigs. Studies have reported that niacin can promote fat catabolism by upregulating the expression of genes involved in mitochondrial fatty acid catabolism in skeletal muscle (24) and reducing the synthesis of triacylglycerol and the expression of perilipin in adipocytes (25). Niacin is an important fat regulator, and the fat deposition in Ningxiang pigs decreased significantly. The additional supplementation of niacin enables Ningxiang pigs to obtain a good lipid-lowering effect, which is sufficient to support more lean meat deposition and improve the lean meat rate. Moreover, the addition of niacin did not appear to have a significant effect on meat quality. Similar findings have been reported in previous studies (26). In future, the effect of niacin on meat quality needs to be explored through gradient experiments to find the appropriate niacin requirement.

The liver is one of the major lipid metabolism organs in animals. On the one hand, the liver can absorb dietary glucose and circulating chylomicron residues for storage through further conversion into triglycerides. On the other hand, the liver can also package lipids into lipoproteins for use by peripheral tissues (27). In this study, about 43.75% of the differential metabolites in the liver were related to lipids. Glycerophospholipids are the main components of cell membranes, and Hishikawa et al. revealed that changes in glycerophospholipid levels can reflect changes in cell membrane composition and permeability (28), which play key roles in cell proliferation, apoptosis, and differentiation (29). Fatty acyl is one of the most basic classes of biological lipids, which represent the main components of complex lipids. As such, fatty acyls play an important role in hypnosis, promoting angiogenesis and anti-inflammation (30–33). Prenol lipids play key roles in regulating the processes of aging-related diseases, diabetes, and inflammation, and they are also important regulators of bone health and cardiovascular homeostasis (34–36). In our experiments, the decrease in the fat rate and the increase in the lean meat rate in Ningxiang pigs were caused by changes in dietary niacin dose, accompanied by changes in liver lipids. KEGG enrichment analysis found that most of these differential metabolites had significant effects on cGMP-PKG and cAMP signaling pathways, sphingolipid signaling pathways, regulation of adipocyte lipolysis, and PPAR signaling pathways. In the body, niacin exists primarily in the form of NAD^+^ or NADP^+^, which transmits electrons through the electron transport chain, thereby participating in the regulation of animal physiological functions (5, 37). By increasing the amount of niacin added and improving the transfer efficiency of the electron transport chain, the energy metabolism and lipid decomposition and metabolism process of Ningxiang pigs are strengthened, and the purpose of improving fat deposition in Ningxiang pigs is achieved.

Numerous studies have shown that nutrients are important bridges linking the interaction between the gut microbiota and the host (38, 39). Regulation of gut microbial composition and maintenance of gut microenvironment homeostasis through nutritional intervention is an important means. In this study, the relative abundance of *Streptococcu*s in the experimental group increased, while the level of *Lactobacillus* decreased at the genus level. Studies have shown that *Streptococcus* is associated with carbohydrate metabolism (40, 41). This indicates that niacin can regulate the synthesis and decomposition of energy in Ningxiang pigs to a certain extent. However, research on *Lactobacillus* playing an important role in combating obesity (42) is contrary to the findings of this current study. A possible reason for this dissonance is the compositional difference in the *Lactobacillus* genus. Spearman's correlation analysis showed that *Lactobacillus* was an important factor leading to the increase in fat percentage in Ningxiang pigs. Niacin supplementation promoted a reduction in the abundance of *Lactobacillus* in the colon, which may be an important factor modulating the reduction in fat deposition in Ningxiang pigs.

In conclusion, niacin increased the lean meat rate and decreased the fat rate of Ningxiang pigs by regulating the composition of gut microflora and lipid metabolism. And there is no negative impact on the meat quality of Ningxiang pigs. This also provided an important guarantee for the healthy breeding of Ningxiang pigs.

## Data availability statement

The sequencing data presented in the study are deposited in the Sequence Read Archive (SRA) repository, accession number PRJNA877854 (http://www.ncbi.nlm.nih.gov/bioproject/877854), and further inquiries can be directed to the corresponding authors.

## Ethics statement

The animal study was reviewed and approved by Animal Care and Use Committee of the Hunan Normal University, Changsha City, Hunan, China. Written informed consent was obtained from the owners for the participation of their animals in this study.

## Author contributions

HY was involved in conceptualization, supervision, and funding acquisition. QiyW was involved in funding acquisition, project administration, and writing-review and editing. ZW was involved in investigation and writing-original draft preparation. XZ was involved in visualization. CZ was involved in data curation. QiaW was involved in validation. WZ was involved in format modification. JX contributed to resources. JC was involved in methodology. QH performed formal analysis. YY was involved in writing-review and editing and funding acquisition. All authors contributed to the article and approved the submitted version.

## Funding

This work was supported by Tianjin Synthetic Biotechnology Innovation Capacity Improvement Project (TSBICIP-CXRC-038), Hunan Provincial Key Laboratory of Animal Nutritional Physiology and Metabolic Process Open Fund Projects (ISA2020113), Scientific Research Project of Hunan Education Department (20B369), and Postgraduate Scientific Research Innovation Project of Hunan Province (QL20220114).

## Conflict of interest

QH was employed by Anyou Biotechnology Group Co., Ltd., Taicang, Jiangsu, China. The remaining authors declare that the research was conducted in the absence of any commercial or financial relationships that could be construed as a potential conflict of interest.

## Publisher's note

All claims expressed in this article are solely those of the authors and do not necessarily represent those of their affiliated organizations, or those of the publisher, the editors and the reviewers. Any product that may be evaluated in this article, or claim that may be made by its manufacturer, is not guaranteed or endorsed by the publisher.
